# Heat-Treated Paraprobiotic *Latilactobacillus sakei* KU15041 and *Latilactobacillus curvatus* KU15003 Show an Antioxidant and Immunostimulatory Effect

**DOI:** 10.4014/jmb.2309.09007

**Published:** 2023-10-05

**Authors:** Jun-Hyun Hyun, Im-Kyung Woo, Kee-Tae Kim, Young-Seo Park, Dae-Kyung Kang, Na-Kyoung Lee, Hyun-Dong Paik

**Affiliations:** 1 Department of Food Science and Biotechnology of Animal Resources, Konkuk University, Seoul 05029, Republic of Korea; 2 Research Institute, WithBio Inc., Seoul 05029, Republic of Korea; 3 Department of Food Science and Biotechnology, Gachon University, Seongnam 13120, Republic of Korea; 4 Department of Animal Biotechnology, Dankook University, Cheonan 31116, Republic of Korea

**Keywords:** Antioxidant effect, immunostimulatory effect, paraprobiotics, *Latilactobacillus sakei*, *Latilactobacillus curvatus*

## Abstract

The lactic acid bacteria, including *Latilactobacillus sakei* and *Latilactobacillus curvatus*, have been widely studied for their preventive and therapeutic effects. In this study, the underlying mechanism of action for the antioxidant and immunostimulatory effects of two strains of heat-treated paraprobiotics was examined. Heat-treated *L. sakei* KU15041 and *L. curvatus* KU15003 showed higher radical scavenging activity in both the 2-azinobis-(3-ethylbenzothiazoline-6-sulfonic acid (ABTS) and 2,2-diphenyl-1-picryl-hydrazyl (DPPH) assays than the commercial probiotic strain LGG. In addition, treatment with these two strains exhibited immunostimulatory effects in RAW 264.7 macrophages, with *L. curvatus* KU15003 showing a slightly higher effect. Additionally, they promoted phagocytosis and NO production in RAW 264.7 cells without any cytotoxicity. Moreover, the expression of tumor necrosis factor-α, interleukin (IL)-1β, and IL-6 was upregulated. These strains resulted in an increased expression of inducible nitric oxide synthase and cyclooxygenase-2. Moreover, the nuclear factor-κB and mitogen-activated protein kinase signaling pathways were stimulated by these strains. These findings suggest the potential of using *L. sakei* KU15041 and *L. curvatus* KU15003 in food or by themselves as probiotics with antioxidant and immune-enhancing properties.

## Introduction 

A robust immune system protects the body against a wide range of diseases and infections; therefore, maintaining a healthy immune system has been emphasized for decades [1]. Conversely, oxidative stress is well known to affect the immune system through free radicals generation [2]. Therefore, immune-enhancing and antioxidant capabilities are closely related to each other [3].

Macrophages, which are phagocytic cells of the innate immune system, play a crucial role in adaptive and innate immune responses to invading antigens [4]. Phagocytic activity is the primary immune response of macrophages against pathogen exposure [5]. When macrophages are activated, they synthesize many inflammatory mediators and cytokines such as nitric oxide (NO), which produce by the action of inducible nitric oxide synthetase (iNOS), tumor necrosis factor (TNF)-α, interleukin (IL)-6, and IL-1β. Additionally, the production of these metabolites is affected by the cellular signaling pathways that follows [6]. The production of NO, TNF-α, IL-6, and IL-1β is regulated by the activation of the mitogen-activated protein kinase (MAPK) signaling pathway, which includes ERK 1/2, JNK, and p38 [7]. In addition, external or internal stimuli may accelerate the NF-κB translocation to the nucleus [8].

Probiotics are live microorganisms that provide health benefits to the host when administered in appropriate amounts [9]. *Latilactobacillus sakei* is abundant in vegetable sources, and its role in sausage fermentation and in the storage of meat products has been recently reported [10]. *Latilactobacillus curvatus* belongs to the *L. sakei* group as a facultative heterofermentative bacterium [11]. Probiotics have been widely studied for their anti-inflammatory, anti-cancer, and immunostimulatory effects [12]. Among them, the immunostimulatory activity of *Lactobacillus* strains is attracting a lot of attention along with the coronavirus issue [13]. Paraprobiotics are bacterial cells that have been rendered inactive through treatments including heat, pressure, sonication, and radiation, have been demonstrated to have the same functional properties as probiotics [14, 15].

*L. sakei* KU15041 and *L. curvatus* KU15003 was selected using the NO production through previous study. Here, the antioxidant and immunostimulatory effects of the heat-treated paraprobiotics *L. sakei* KU15041 and *L. curvatus* KU15003 on RAW 264.7 macrophages were examined. In order to better understand these processes, production of NO and cytokines, phagocytic activity, and activation of cellular signaling pathways such as MAPKs and NF-κB were investigated.

## Material and Methods

### Paraprobiotic Sample Preparation

*L. sakei* KU15041 and *L. curvatus* KU15003 were isolated from kimchi, a traditional Korean food. *L. rhamnosus* GG (LGG), a reference bacterial strain, was obtained from the Korean Collection for Type Culture (KCTC), Korea. The bacterial strains were initially cultured in de Man, Rogosa, and Sharpe (MRS) broth (Difco, BD Biosciences, USA) at 37°C for 22 h. After two subcultures in MRS medium, the bacterial strains were subjected to heat treatment at 80°C for 30 min in a water bath. Subsequently, the samples were centrifuged at 12,000 ×*g* for 5 min and washed twice with phosphate-buffered saline (PBS; Hyclone, USA) under the same centrifugation conditions. The collected bacterial cells were suspended either in PBS or Dulbeccós modified Eaglés medium (DMEM; Hyclone) at concentrations of 7 or 9 log CFU/ml.

### RAW 264.7 Cell Culture

RAW 264.7 cells were obtained from the Korean Cell Line Bank (KCLB), Korea, and were cultured in DMEM with 10% fetal bovine serum (Life Technologies, USA) and 1% penicillin-streptomycin (Hyclone). Cells were used for experiments once they reached 80% confluence after subculturing in a 5% CO_2_ and 37°C incubator (MCO-18AIC, Sanyo Co., Japan).

### ABTS and DPPH Radical Scavenging Assays

The antioxidant potential of heat-treated lactic acid bacteria (LAB) strains was assessed using 2,2'-azino-bis(3-ethylbenzothiazoline-6-sulfonic acid) diammonium salt (ABTS) radical scavenging and 1,1-Diphenyl-2-picryl-hydrazyl radical (DPPH) assays. The ABTS radical scavenging activity was measured using a modified method described by Choi *et al*. [16]. Briefly, a solution of 14 mM ABTS and 5 mM potassium persulfate was prepared in potassium phosphate buffer (pH 7.4) and left overnight in the dark. The absorbance of ABTS solution was adjusted to 0.7 ± 0.02 at 734 nm immediately before the experiment. A mixture of 900 μl of ABTS solution and 100 μl of the paraprobiotic sample (9 log CFU/ml) was incubated for 15 min in the dark. The absorbance of the supernatant was measured at 734 nm after centrifugation at 14,000 ×*g* for 1 min. The ABTS radical scavenging activity was calculated using the following formula:

ABTS radical scavenging activity (%) = (1 – A_sample_/A_control_) × 100

Where A_sample_ and A_control_ represent the absorbance values of the sample and control (PBS), respectively.

The DPPH radical scavenging activity was determined using a modified method described by Song *et al*. [17]. In brief, a mixture of 500 μl of 0.1 mM DPPH solution (in 99% ethanol) and an equal volume of paraprobiotic sample (9 log CFU/ml) was incubated for 30 min in the dark. Subsequently, the mixtures were centrifuged at 14,000 ×*g* for 1 min and the absorbance of the supernatant was measured at 517 nm using a spectrometer. DPPH radical scavenging activity was calculated using the following formula:

DPPH radical scavenging activity (%) = (1 – A_sample_/A_control_) × 100

Where A_sample_ and A_control_ represent the absorbance values of the sample and control (PBS), respectively.

### Cell Viability and NO Assay

Cell viability was assessed using the 3-(4,5-dimethylthiazol-2-yl)-2,5-diphenyltetrazolium bromide (MTT) assay as described by Hyun *et al*. [18]. Briefly, RAW 264.7 cells (2 × 10^5^ cells/well) were seeded into a 96-well plate for 4 h. After pre-incubation, the cells were treated with either 7 log CFU/ml of paraprobiotic samples or 10 ng/ml lipopolysaccharide (LPS) for 24 h. After collecting 100 μl of supernatant for the NO assay, 25 μl of MTT reagent (2.5 mg/ml in PBS) was added to each well and incubated for 40 min. Subsequently, the solution of each well was aspirated, and 200 μl of dimethyl sulfoxide (DMSO) was added to dissolve the formazan deposits. Absorbance was measured at 570 nm, and cell viability was determined as a percentage relative to the negative control group.

To determine the NO production, 100 μl of supernatant from the MTT assay was transferred to a second 96-well plate. Next, 100 μl of Griess reagent was added and incubated for 15 min at room temperature in the dark. After incubation, NO production was measured at 540 nm using a standard curve of sodium nitrite in DMEM.

### Phagocytosis Activity

The phagocytic ability of the heat-treated bacterial samples was evaluated using the neutral red uptake assay based on the method described by Lee *et al*. [19], with slight modifications. RAW 264.7 cells (2 × 10^5^ cells/well) were pre-incubated in 24-well plates for 4 h. Then, 7 log CFU/ml of paraprobiotic samples or LPS (10 ng/ml) was added to each well and incubated for another 24 h. After removing the supernatant, a 0.075% neutral red solution (in PBS) was added and incubated for 1 h. To dissolve the neutral red, the wells were washed three times with PBS, and a lysis solution (acetic acid and ethanol at a 1:1 ratio) was added. The absorbance was measured at 540 nm and phagocytic activity was determined as a percentage absorbance relative to that of the negative control group.

### Production of TNF-α, IL-1β, and IL-6

The production of TNF-α, IL-1β, and IL-6 was assessed using an ELISA kit (Thermo Fisher Scientific, USA). Briefly, RAW 264.7 cells were incubated for 4 h in a 12-well plate. After pre-incubation, RAW 264.7 cells were exposed to paraprobiotic samples (7 log CFU/ml) or LPS (10 ng/ml) for 24 h. The supernatant was stored at –18°C, and then diluted in DMEM media appropriately before being used in the experiment. Levels of the three cytokines were determined according to the manufacturer’s instructions.

### Western Blot Analysis

The protein expression levels of iNOS and COX-2, as well as NF-κB and MAPK signaling pathway activation in RAW 264.7 cells, were assessed using western blot analysis [20] with some modifications. For iNOS and COX-2 expression, RAW 264.7 cells (1 × 10^6^ cells/ml) were incubated for 16 h in 60-mm culture dishes and then treated with paraprobiotic samples (7 log CFU/ml) or LPS (10 ng/ml) for additional 24 h. To analyze the NF-κB and MAPK signaling pathways, RAW 264.7 cells (2 × 10^6^ cells/ml) were seeded for 16 h and treated with paraprobiotic samples (7 log CFU/ml) or LPS (10 ng/ml) for specified time periods.

For western blot analysis, the cells of each treatment were washed twice with ice-cold PBS and lysed using Pro-prep lysis buffer (iNtRON Biotechnology, Korea) with protease and phosphatase inhibitors. The cell lysates (20 μg) were resolved by 12% SDS-PAGE following quantification with a DC Protein Assay Kit (Bio-Rad, USA), and transferred to polyvinylidene difluoride membranes (Bio-Rad). Membranes were blocked with 5% skim milk in TBS-T for 1 h, followed by overnight incubation at 4°C with primary antibodies. Horseradish peroxidase-conjugated secondary antibodies were applied for 2 h at room temperature after TBS-T washes. Protein bands were visualized using an enhanced chemiluminescence detection kit (Bio-Rad) and X-ray film, and quantified with ImageJ software (National Institutes of Health, USA).

### Statistical Analysis

Each experimental data is the mean ± S.D of triplicates. Statistical analyses were performed using IBM SPSS version 18.0 software (SPSS Inc., USA). One-way analysis of variance (ANOVA) followed by Duncan's multiple range test was used for statistical comparisons.

## Results

### Antioxidant Effects of the Paraprobiotics *L. sakei* KU15041 and *L. curvatus* KU15003

ABTS and DPPH assays were conducted to verify the antioxidant effects of the paraprobiotics *L. sakei* KU15041 and *L. curvatus* KU15003 (Table 1). In the ABTS assay, LGG showed 20.56% ABTS radical scavenging activity, whereas *L. sakei* KU15041 and *L. curvatus* KU15003 exhibited high ABTS radical scavenging activity of 34.73%and 35.30%, respectively.

In the DPPH assay, heat-treated LGG showed 11.30% DPPH radical scavenging activity. *L. sakei* KU15041 and *L. curvatus* KU15003 exhibited higher DPPH radical-scavenging activities than LGG (15.29% and 18.84 %, respectively), with *L. curvatus* KU15003 showing the highest DPPH radical scavenging activity. These results suggest that *L. sakei* KU15041 and *L. curvatus* KU15003 have high antioxidant potentials.

### NO Production and Phagocytic Activity without Cell Cytotoxicity

**Fig. 1:**
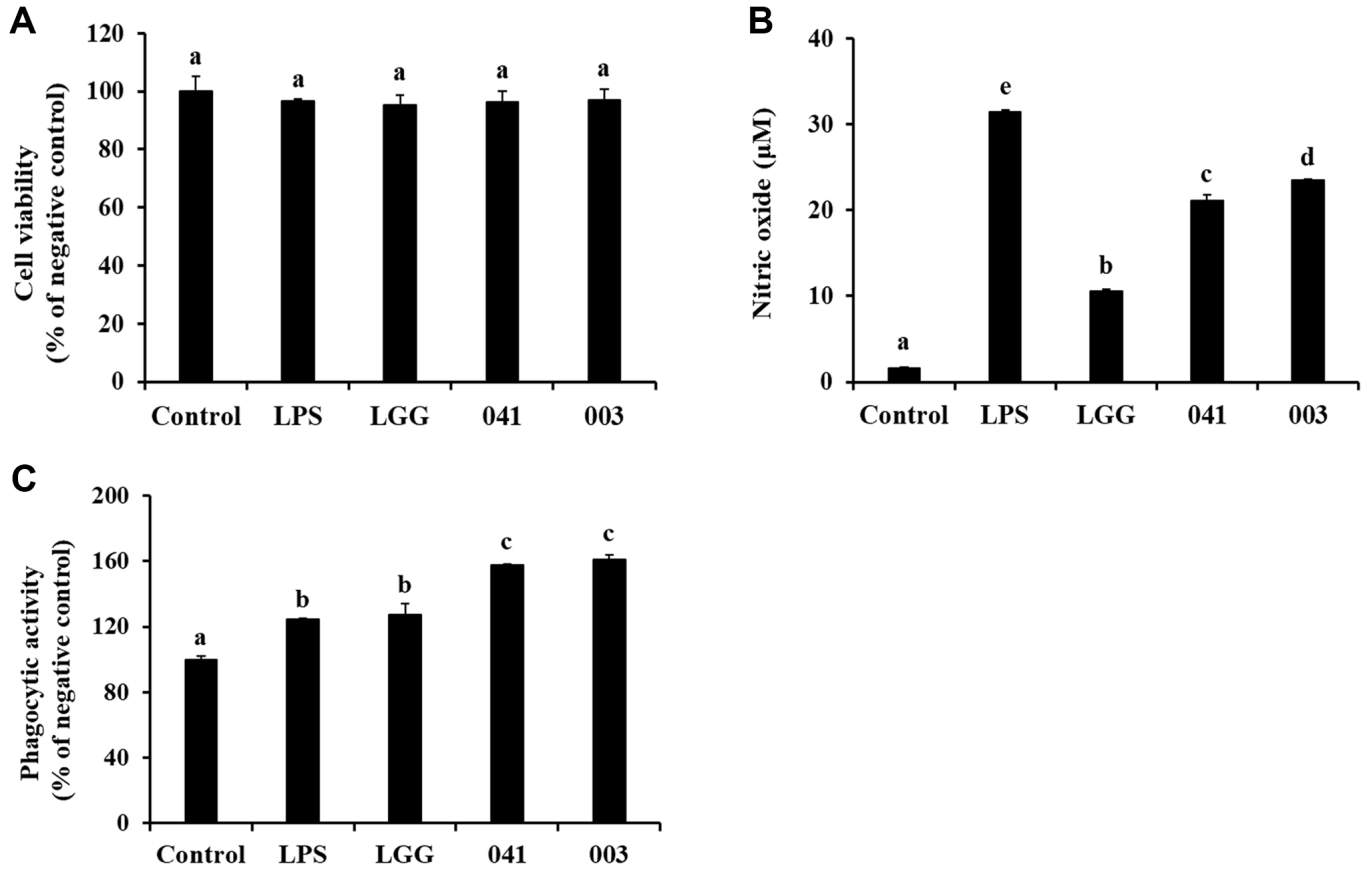
Cell viability (A), nitric oxide (NO) production (B), and phagocytic activity (C) of paraprobiotic *Lactobacillus* strains in RAW 264.7 cells. Cells were preincubated for 4 h, and treated with LPS (10 ng/ml) or heat-treated bacterial sample for 24 h. Control, nontreated LPS; LPS, LPS treatment; LGG, *L. rhamnosus* GG; 041, *L. sakei* KU15041; 003, *L. curvatus* KU15003. Different letters mean significant differences between the groups of samples (*p* < 0.05).

To determine the immunostimulatory potential of the paraprobiotics *L. sakei* KU15041 and *L. curvatus* KU15003, Griess and Neutral Red uptake assays were conducted. An MTT assay was also performed to examine the cytotoxic effects of the paraprobiotic samples. As shown in Fig. 1A, there was no noticeable difference in cell viability between the LPS (10 ng/ml) treated group and any of the paraprobiotic treated groups compared to the control group. These results suggested that the 7 log CFU/mL bacterial concentration used in the experiment did not have any considerable impact on the cells. The paraprobiotic samples showed increased NO production. Both *L. sakei* KU15041 and *L. curvatus* KU15003 produced more NO than the commercial reference strain LGG (Fig. 1B). As shown in Fig. 1C, the paraprobiotics *L. sakei* KU15041 and *L. curvatus* KU15003 exhibited enhanced phagocytic activity. These results suggest that the paraprobiotic samples have immunostimulatory potential by increasing NO production and phagocytic activity without cell cytotoxicity.

### Production of TNF-α, IL-6, and IL-1β

**Fig. 2:**
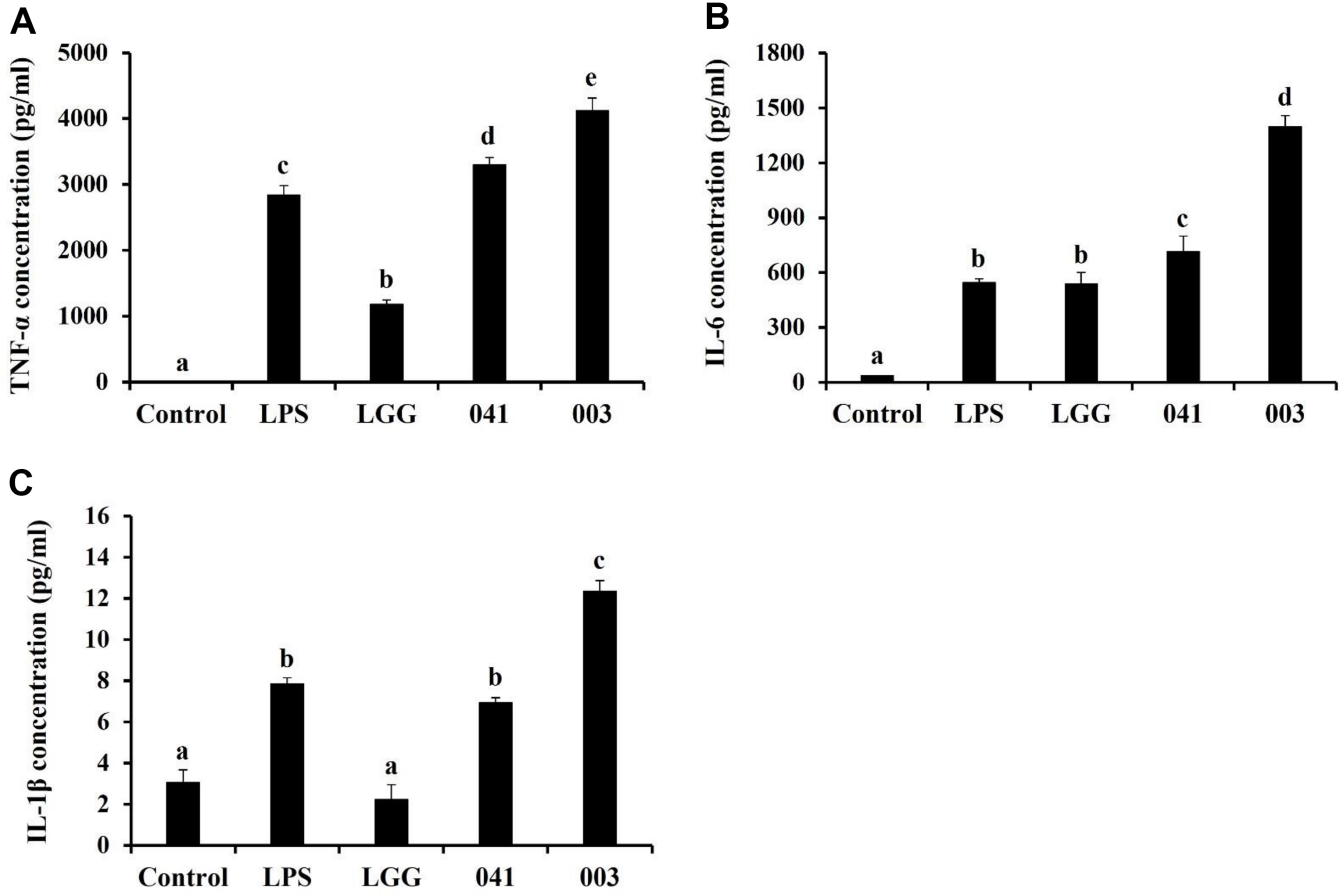
Concentration of TNF-α (A), IL-6 (B), and IL-1β (C) studied using ELISA in RAW 264.7 cells following treatment with paraprobiotic *Lactobacillus* strains. Cells were preincubated for 4 h, and treated with LPS (10 ng/ml) or heat-treated bacterial sample for 24 h. Control, nontreated LPS; LPS, LPS treatment; LGG, *L. rhamnosus* GG; 041, *L. sakei* KU15041; 003, *L. curvatus* KU15003. Different letters mean significant differences between the groups of samples (*p* < 0.05).

TNF-α, IL-6, and IL-1β production was assessed using ELISA kits to determine the immunostimulatory effects of paraprobiotic samples. TNF-α, IL-6, and IL-1β levels are shown in Fig. 2. Treatment with LGG showed lower TNF-α, IL-6, and IL-1β production compared to the 10 ng/ml LPS-treated group (Fig. 2). However, after treatment of *L. sakei* KU15041 and *L. curvatus* KU15003, the production of these three cytokines was upregulated and exhibited levels that were similar or even higher compared to the LPS (10 ng/ml) group. Furthermore, *L. curvatus* KU15003 treatment was more effective than other treatments. These findings suggest that *L. sakei* KU15041 and *L. curvatus* KU15003 are potential immunomodulatory agents.

### Expression of iNOS and COX-2

**Fig. 3:**
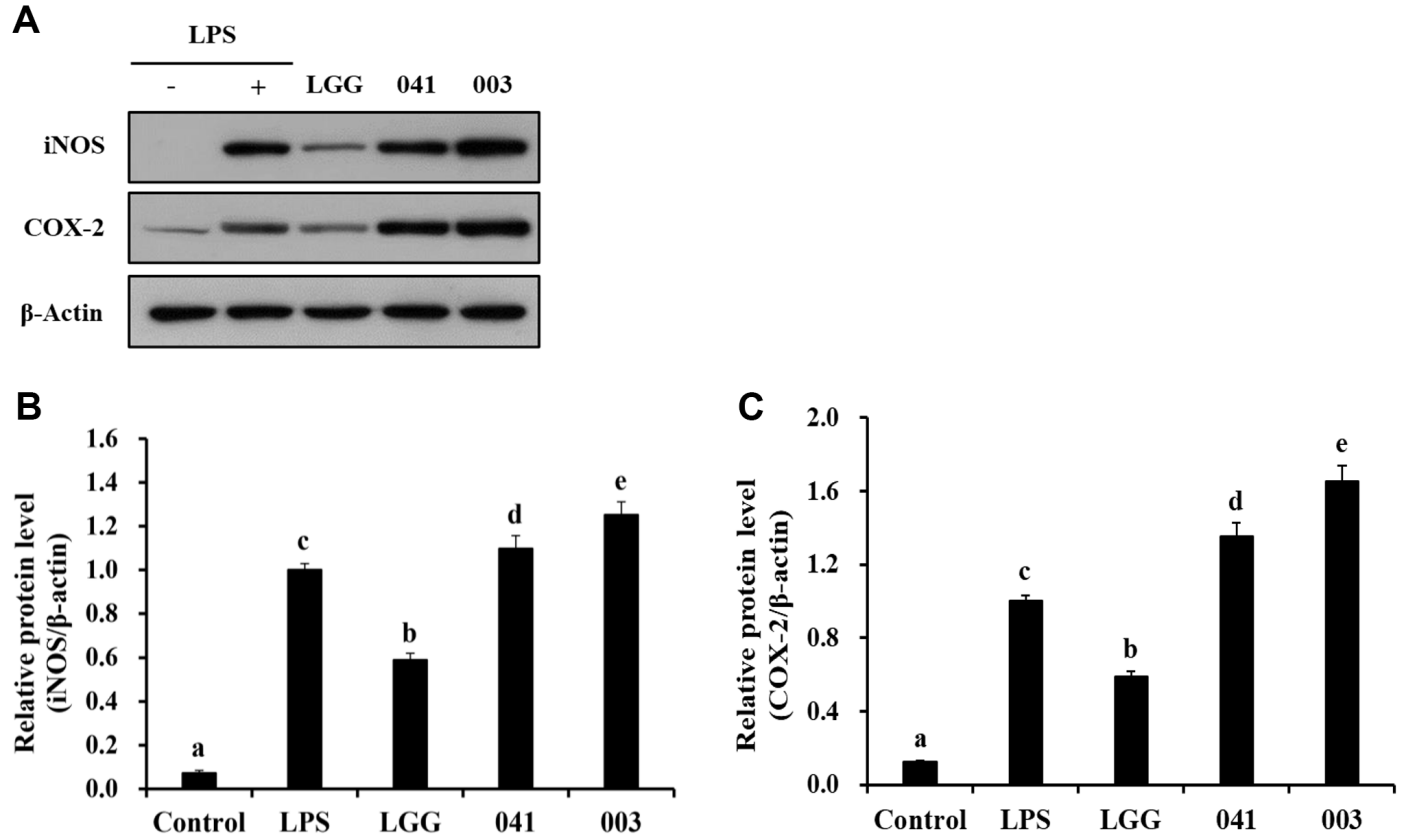
Western blot analysis of iNOS and COX-2 (A), quantification of iNOS/ β-actin (B), and COX-2/β- actin (C) via imageJ observed in RAW 264.7 cells following treatment with paraprobiotic *Lactobacillus* strains. β-Actin was employed as an internal loading control. Cells were treated with LPS (10 ng/ml) or heat-treated bacterial sample for 24 h. Control, nontreated LPS; LPS, LPS treatment; LGG, *L. rhamnosus* GG; 041, *L. sakei* KU15041; 003, *L. curvatus* KU15003. Different letters mean significant differences between the groups of samples (*p* < 0.05).

Western blotting was performed to investigate the effects of *L. sakei* KU15041 and *L. curvatus* KU15003 on iNOS and COX-2 expression in macrophages. The cells were treated with 10 ng/mL LPS or paraprobiotic samples, and untreated cells were used as negative controls. As shown in Fig. 3, treatment with the paraprobiotic samples enhanced the protein levels of iNOS and COX-2. Additionally, the expression of iNOS and COX-2 in RAW 264.7 macrophages was significantly increased by *L. sakei* KU15041 and *L. curvatus* KU15003 compared to the control group (Fig. 3A and 3B). These findings indicate that the paraprobiotic samples have the potential to augment immune responses by inducing iNOS and COX-2 in RAW 264.7 macrophages.

### Protein Expression Levels of NF-κB Signaling Pathways

**Fig. 4:**
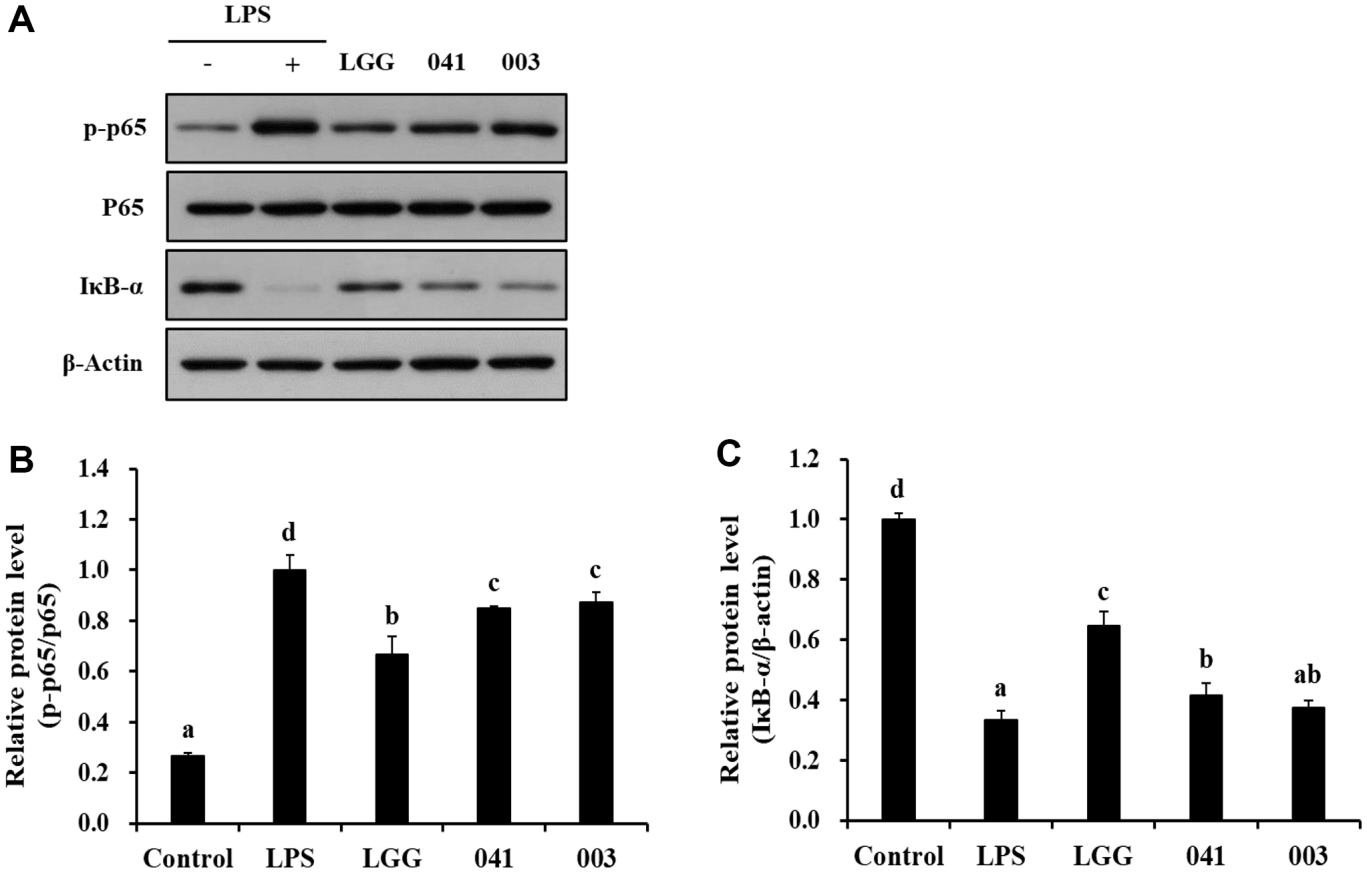
Western blot analysis of NF-κB signaling pathway factors (A), quantification of p-p65/p65 (B), and IκB-α/β-actin (C) via imageJ observed in RAW 264.7 cells following treatment with paraprobiotic *Lactobacillus* strains. β-Actin was employed as an internal loading control. Cells were treated with LPS (10 ng/ml) or heattreated bacterial sample for 30 min. Control, nontreated LPS; LPS, LPS treatment; LGG, *L. rhamnosus* GG; 041, *L. sakei* KU15041; 003, *L. curvatus* KU15003. Different letters mean significant differences between the groups of samples (*p* < 0.05).

To examine whether the paraprobiotics demonstrated their immunostimulatory abilities through cellular signaling pathways, the activation of NF-κB signaling pathway was assessed using western blot analysis. The results showed that the treatment with the heat-treated bacterial sample induced the activation of NF-κB signaling by elevation the p-p65 expression and degradation of IκB-α (Fig. 4). In RAW 264.7 macrophages, the expression of p-p65 was increased following treatment with *L. sakei* KU15041 and *L. curvatus* KU15003 compared to that of the control group (Fig. 4A). Both strains exhibited similar levels of p-p65 expression, indicating their potential to activate signaling pathways.

Furthermore, the expression of IκB-α was decreased in RAW 264.7 macrophages treated with the paraprobiotic samples, displaying cellular differentiation and activation of the immune response (Fig. 4B). Interestingly, these two strains showed significant activation of the NF-κB signaling pathway compared to the commercial strain LGG. These findings suggest that the paraprobiotic *L. sakei* and *L. curvatus* strains may improve the immune response by activating cellular signaling pathways.

### Protein Expression Levels of MAPK Signaling Pathways

**Fig. 5:**
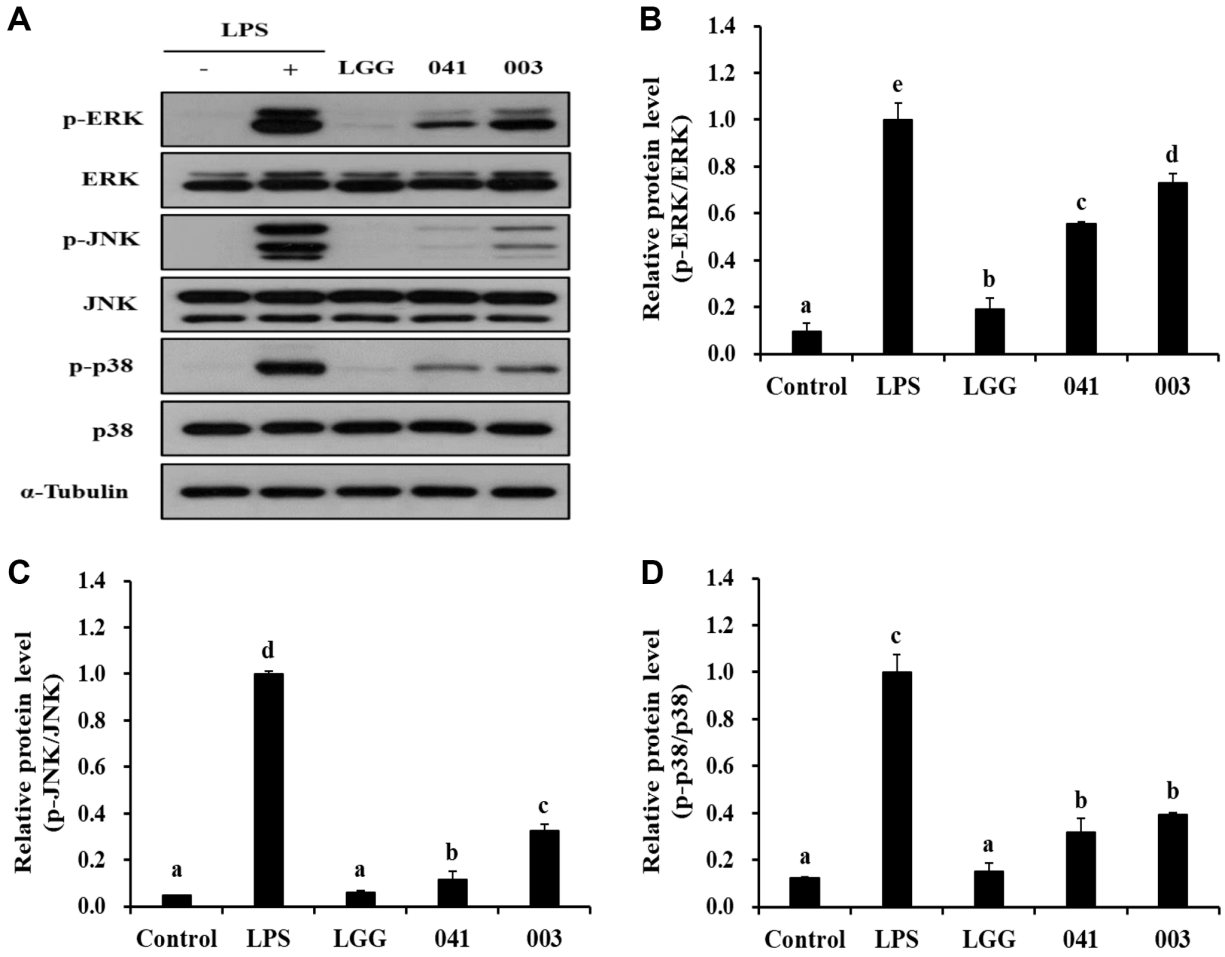
Western blot analysis of MAPK signaling pathway factors (A), quantification of p-ERK/ERK (B), p-JNK/ JNK (C), and p-p38/p38 (D) via imageJ observed in RAW 264.7 cells following treatment with paraprobiotic *Lactobacillus* strains. α-Tubulin was employed as an internal loading control. Cells were treated with LPS (10 ng/ml) or heat-treated bacterial sample for 30 min. Control, nontreated LPS; LPS, LPS treatment; LGG, *L. rhamnosus* GG; 041, *L. sakei* KU15041; 003, *L. curvatus* KU15003. Different letters mean significant differences between the groups of samples (*p* < 0.05).

To investigate whether the paraprobiotic samples affected cellular signaling pathways related to immune enhancement, western blotting was performed to examine the MAPK signaling pathway. Specifically, we focused on three factors, p38, ERK, and JNK (Fig. 5). However, the expression of p-ERK did not significantly increase after treatment with LGG; however, treatment with *L. sakei* KU15041 and *L. curvatus* KU15003 resulted in increased expression, with *L. curvatus* KU15003 showing the most effective results (Fig. 5A). Although p-JNK expression levels did not show a considerable increase in any of the paraprobiotic treated samples, there was a definite increase observed in the case of *L. curvatus* KU15003 treated samples (Fig. 5B). In addition, p-p38 expression did not show much difference with LGG, but *L. sakei* KU15041 and *L. curvatus* KU15003 treatments increased p-p38 expression levels. Based on these results, it can be inferred that the paraprobiotic samples activate the MAPK signaling pathway, thereby exerting immunostimulatory effects.

## Discussion

The immune system plays a critical role in defending the body against various pathogens and maintaining homeostasis [21]. Macrophages are primary immune cells that recognize and engulf invading microorganisms through phagocytosis, and initiate a series of signaling pathways to activate immune responses [22]. However, excessive immune activation can lead to tissue damage and chronic inflammation, highlighting the importance of balancing immune responses [23]. Nevertheless, a well-controlled immune response is crucial to protecting the host from many infectious agents. Therefore, improving the ability of the immune system to fight pathogenic threats while maintaining a controlled immune response is an essential goal in modern immunological research [24, 25]. Moreover, antioxidant capacity is closely associated with immunostimulatory potential because the antioxidant defense system used to combat oxidative stress also affects the immune system [26].

Probiotics have been studied for their various functions and potential health benefits, including antioxidant, immunostimulatory, and anti-inflammatory effects [27]. Bacterial strains exert immunomodulatory effects by interacting with the host immune system. Furthermore, probiotics can directly interact with specific receptors on immune cells, including macrophages, dendritic cells, and lymphocytes, to stimulate immune responses [28, 29]. Paraprobiotics are inactive probiotics that maintain their biofunctionality, exhibit increased shelf life, and are often more efficient in the application of functional foods compared to probiotics [30]. According to previous research, paraprobiotics, especially heat-killed bacteria, are considered safe alternatives to live probiotics and have shown potential in managing gastrointestinal disorders and supporting immune health [31]. Moreover, both cell wall components and exopolysaccharides (EPS) in probiotics can retain their functionality, even in heat-treated paraprobiotic forms [15]. Peptidoglycans and lipopolysaccharides are stable under heat treatment and can interact with and modulate immune responses. Similarly, EPS produced by probiotic bacteria can maintain its antioxidant and immunomodulatory properties even after processing [32].

Oxidative stress may be inhibited by the scavenging of ABTS and DPPH free radicals [33]. LGG, a commercial probiotic strain, exhibits outstanding radical scavenging activity [34]. In contrast, *L. sakei* KU15041 and *L. curvatus* KU15003 exhibited higher ABTS and DPPH free radical scavenging activities than LGG (Table 1).

NO production and phagocytic activity provide important insights into the immunomodulatory effects of LAB. NO, a crucial mediator of immune modulation, is produced by immune cells and plays a key role in the host’s defense against infection [35]. The phagocytic activity of macrophages is a fundamental mechanism in host defense against infectious agents and in inflammation and immune responses [36]. Treatment with 7 log CFU/ml of heat-treated *L. sakei* KU15041 and *L. curvatus* KU15003 significantly increased NO production, especially for *L. curvatus* KU15003 treatment, indicating the activation of immune responses (Fig. 1B). Furthermore, the phagocytic activity of RAW 264.7 macrophages was enhanced by treatment of heat-treated bacterial samples without causing cell cytotoxicity (Fig. 1A and 1C). These findings suggest that heat-treated bacterial samples stimulate macrophages by increasing NO production and phagocytic activity.

Cytokines have been studied to coordinate the immune response and mediate intracellular communication [37]. Moreover, macrophages secrete cytokines, such as TNF-α, IL-6, and IL-1β which play an important role in initiating the immune response [38]. As shown in Fig. 2, the paraprobiotics *L. sakei* KU15041 and *L. curvatus* KU15003 upregulated the concentrations of these three cytokines. These results imply that the heat-treated bacterial samples modulate immune responses by influencing cytokine production. iNOS plays a crucial role in the synthesis of NO, which serves as a mediator in diverse immune and pro-inflammatory responses of macrophages [39]. Additionally, macrophages stimulation, induces COX-2 expression by cytokines including TNF-α and IL-1β [40]. Fig. 3 shows that the expression of iNOS and COX-2 is effectively increased following treatment of *L. sakei* KU15041 and *L. curvatus* KU15003 in RAW 264.7 cells compared to LGG treatment. This study demonstrated the immunostimulatory effects of heat-treated paraprobiotics through the regulation of iNOS and COX-2 expression.

The modulation of immune responses by probiotics involves the regulation of cellular signaling pathways such as NF-κB and MAPK signaling pathways. Activation of the NF-κB signaling pathway triggers the production of pro-inflammatory cytokines, and antimicrobial peptides [41, 42]. Phosphorylation of p65, as a NF-κB subunit, promotes the degradation of IκB-α and activation of NF-κB [43]. Similarly, it is well known that the immune response is activated through the MAPK signaling pathway, including ERK, JNK, and p38, which are involved in immune cell activation, cytokine production, and immune regulation [44]. As shown in Fig. 4, heat-treated bacterial samples showed upregulated p-p65 expression compared to the control group, and *L. curvatus* KU15003 induced the degradation of IκB-α. Accordingly, these results suggest that the heat-treated bacterial samples enhance the immune response in macrophages through the activation of NF-κB signaling pathway. Compared to the control group, treatment with heat-treated bacterial samples activated MAPK signaling pathways by affecting the expression levels of p-ERK, and p-p38. *L. curvatus* KU15003 also showed a noticeable role in activating p-JNK expression (Fig. 5). The heat-treated paraprobiotic samples used in this experiment seemed to have worked more effectively on NF-κB signaling than on the MAPK signaling pathway.

In summary, the paraprobiotics *L. sakei* KU15041 and *L. curvatus* KU15003 showed antioxidant potential and immunostimulatory effects in RAW 264.7 macrophages. Heat-treated *L. sakei* KU15041 and *L. curvatus* KU15003 exhibited prominent antioxidant potential through ABTS and DPPH radical-scavenging activities. Moreover, treatment with heat-treated bacterial samples increased NO production and phagocytic activity, and upregulated the expression of iNOS and COX-2. In addition, the production of cytokines such as TNF-α, IL-6, and IL-1β was modulated by the paraprobiotic samples. Furthermore, the paraprobiotic samples stimulated NF-κB and MAPK cell signaling pathways in RAW 264.7 macrophages. The discovery of novel functionalities in paraprobiotic samples is of great significance and proof that these heat-treated bacterial samples have potential as probiotic ingredients for improving antioxidant potential and immune function. To investigate additional applications and mechanisms of action, additional studies, analyses of paraprobiotic components, and animal experiments should be warranted.

## Figures and Tables

**Table 1:** Antioxidant activity of paraprobiotic *Lactobacillus* strains.

Antioxidant assay	Radical scavenging activity (%)		
	*L. rhamnosus* GG	*L. sakei* KU15041	*L. curvatus* KU15003
ABTS assay	20.56 ± 1.02^a^	34.73 ± 2.49^b^	35.30 ± 2.10^b^
DPPH assay	11.30 ± 1.24^a^	15.29 ± 0.10^b^	18.84 ± 1.18^c^

Different letter indices indicate significant differences between groups (*p* < 0.05).
